# Is There Still a Role of Plasma Exchange in the Current Management of ANCA-Associated Vasculitides?

**DOI:** 10.1007/s11926-022-01064-8

**Published:** 2022-03-22

**Authors:** Task Toyoda, Max Yates, Richard A. Watts

**Affiliations:** 1 Norwich Medical School, University of East Anglia, Norwich, Norfolk, NR4 7TJ UK; 2 Norfolk and Norwich University Hospital, Norwich, UK

**Keywords:** Plasma exchange, ANCA-associated vasculitis, End-stage renal failure, Diffuse alveolar haemorrhage

## Abstract

**Purpose of Review:**

Plasma exchange (PLEX) is often recommended as an adjunctive therapy for patients with ANCA-associated vasculitis (AAV) in the setting of rapidly progressive glomerulonephritis or diffuse alveolar haemorrhage. Since ANCAs are pathogenic, it seems a reasonable and justified approach to remove them through therapeutic PLEX, as despite advances in immunosuppressive therapy regimens, AAV is associated with significant morbidity and death. However, the association between ANCA levels and mortality or disease activity is uncertain. In addition, any treatment must be judged on the potential risks and benefits of its use. Here, we summarise the current data on PLEX usage in patients with AAV.

**Recent Findings:**

The largest randomised trial to date the Plasma Exchange and Glucocorticoids in Severe ANCA-Associated Vasculitis (PEXIVAS) study failed to show added benefit for PLEX on the prevention of death or end-stage renal failure (ESRF) for the management of patients with severe AAV. However, there is a possibility that PLEX delays dialysis dependence and ESRF in the early stages of the disease. Regardless of whether this is only for 3 to 12 months, this could be of clinical significance and a substantial improvement in patient’s quality of life.

**Summary:**

Cost utility analysis and trials including patient-centred outcomes are required to evaluate the use of PLEX. Furthermore, ascertaining those at high risk of developing ESRF could help identify those who may benefit from PLEX the most, and further insights are required in setting of diffuse alveolar haemorrhage.

## Introduction

Antineutrophil cytoplasmic antibody (ANCA)–associated vasculitis (AAV) encompasses a group of rare systemic inflammatory disorders which affect the small arterial vessels, commonly of the renal and respiratory tract. These include granulomatosis with polyangiitis (GPA), microscopic polyangiitis (MPA) and eosinophilic granulomatosis with polyangiitis (EGPA) and have a combined incidence of around 20 per million per year [1]. The Chapel Hill Consensus nomenclature incorporates clinical and immunological features [2]. They are often associated with high levels of ANCA directed against proteinase 3 (PR3) and/or myeloperoxidase (MPO) in the cytoplasm of neutrophils [3].

The current standard of care for induction of remission is a combination therapy comprising high-dose glucocorticoids with either cyclophosphamide or rituximab [4]. This can induce remission in up to 90% of patients, yet the mortality of AAV remains disproportionately high reported at 19.5% at 1 year [5, 6••, 7]. The significant mortality burden on AAV patients is multifactorial and is mostly attributable to active vasculitis, renal impairment and treatment-related adverse events, particularly infection secondary to immunosuppression [5]. In severe cases of AAV, usually presenting with rapidly progressive glomerulonephritis and/or severe diffuse alveolar haemorrhage (DAH), plasma exchange (PLEX) is often recommended as an adjunctive therapy [8].

PLEX is a therapeutic treatment involving the removal of serum plasma through centrifugation, or filtration, to remove pathogenic substances such as immunoglobulins [9]. As large volumes are apheresed, patients are often replenished with either fresh frozen plasma or human albumin preparation. PLEX is indicated in a spectrum of diseases such as autoimmune, neurological, haematological and renal disorders [10]. Complications of PLEX include adverse reactions to human albumin solution or fresh frozen plasma (1.4–20%), hypocalcaemia (1.7–9.1%), hypotension/hypovolaemia (8.4%), death (0.05%), anaphylaxis (0.25%), haemorrhage (0.02%) and infection (0.02%) [11]. Very little on the cost–benefit of PLEX in AAV has been studied. However, a few papers have outlined the cost of PLEX in neurological disease. Depending on the country, it is estimated that the total cost of PLEX is around GBP 2000 or up to USD 4500 and USD 50,000 for the PLEX machine [12, 13]. Nevertheless, it is agreed that PLEX is an expensive therapeutic medium and, therefore, its invasive, time and cost-intensive nature must be balanced with the benefit.

It is widely accepted that ANCAs may play a pathogenic role in AAV pathophysiology [14]. The aetiology of AAV with a production of pathogenic ANCA is unclear and thought to be multifactorial with genetic, immunological and environmental triggers [15]. The ANCA cytokine-sequence theory demonstrated in vitro models has shown activation of neutrophils, monocytes and endothelial cells via IgG MPO- and PR3-ANCA. Priming and apoptosis of neutrophils cause intracellular PR3 and MPO translocation, endovascular adherence, inflammation and eventually necrotising vasculitis [16, 17]. There are several properties of ANCA such as their high molecular weight and long half-life which makes them a suitable target for apheresis [18].

The rapid removal of ANCAs by means of PLEX may reduce organ damage from AAV [19–23]. We know that PLEX is extremely effective in reducing ANCA titres [24••]. However, the association between ANCA levels and mortality or disease activity is uncertain [22, 25]. The use of PLEX for the rapid reduction of antibodies resulting in marked reduction disease burden or faster remission is, to an extent, theoretical [26].

The typical PLEX regimen in AAV is 1–1.5 l of total plasma volume exchanged over 7 sessions over a median period of 14 days [10]. It is debated whether there is a dose-dependent effect of the number of sessions on mortality. Whilst some authors suggest possible improvements in renal function beyond 7 exchanges, other more robust study methods have shown no effect on mortality or end-stage renal failure (ESRF) [23, 27].

## Plasma Exchange in ANCA-Associated Vasculitis

PLEX has been used to treat AAV for several decades [28–30]. The first large scale randomised controlled trial to investigate the role of PLEX in the treatment of AAV was the MEPEX study which enrolled 137 patients with severe renal AAV [31••]. Participants were randomly assigned to receive PLEX or intravenous (IV) methylprednisolone in addition to standard remission therapy. At 3 months, 69% of the PLEX group versus 49% of the IV methylprednisolone group were dialysis-independent (95% confidence interval (CI) 18–35% difference, *p* = 0.02). Furthermore, at 12 months, PLEX was found to have a protective effect on ESRF with a risk reduction of 24% (95% CI 6.1–41.0). However, the median follow-up period of 3.95 years failed to maintain the long-term benefit of renal recovery or mortality (hazard ratio (HR) 0.81, 95% CI 0.53–1.23) [32••]. A meta-analysis of nine studies including 387 patients found a significant effect of PLEX in the pooled reduction of ESRF or death (relative risk (RR) 0.80, 95% CI 0.65–0.99, *p* = 0.04) and ESRF alone (RR 0.64, 95% CI 0.47–0.88, *p* = 0.007) but not death alone (RR 1.00, 95% CI 0.71–1.42) [23]. Considering the strong association between ESRF and mortality, these results could be due to a type 1 error from the combination of smaller trials. The authors also admit that the results were inconclusive due to insufficiently robust sensitivity analyses and lack of randomisation within the cohorts leading to the introduction of bias.

This study formed the basis for the use of PLEX during 2000s to 2010s. The limitation of the study resulted in the development of the Plasma Exchange and Glucocorticoids in Severe ANCA-Associated Vasculitis (PEXIVAS) trial [33]. PEXIVAS is now the largest randomised trial to be conducted in AAV patients. Walsh et al. [33] recruited 704 patients with severe AAV from 95 centres in 16 countries. Patients were randomised to a 2-by-2 factorial design to either PLEX or no PLEX plus standard-dose or reduced-dose glucocorticoids. The primary outcome was a composite endpoint of death or ESRF, and the trial had a median follow-up duration of 2.9 years. Severe AAV was defined as the presence of renal involvement with evidence of glomerulonephritis, an estimated glomerular filtration rate (eGFR) < 50 ml/min/1.73m^2^ or pulmonary haemorrhage. The primary outcome was reached in 100 patients (28.4%) in the PLEX group versus 109 (31.0%) in the control group (HR 0.86, 95% CI 0.65–1.13). Secondary outcomes included all-cause mortality, ESRF, sustained remission, serious adverse events and serious infections at 12 months, none of which showed significant differences.

PEXIVAS showed PLEX had no added benefit on the prevention of death or ESRF in the management of patients with severe AAV. To fully comprehend these findings, it is important to consider the differences with the MEPEX trial [31••]. The PEXIVAS cohort was different; firstly, it encompassed AAV patients with pulmonary haemorrhage, a complication of AAV which carries significant mortality and a subgroup likely to benefit from PLEX. Secondly, the differences in renal characteristics are important to note. The PEXIVAS trial had broader inclusion parameters. Patients were eligible for the trial with an eGFR of < 50 ml/min/1.73^2^, in contrast to MEPEX which included serum creatinine > 500 umol/l or dialysis dependence. Only 29% of patients in the PEXIVAS cohort would have met the renal threshold to be included in the MEPEX trial. The inclusion of patients with less severe renal morbidity may diminish the detection of benefits. However, subgroup analysis of patients with serum creatinine > 500 umol/l or requiring dialysis showed no difference (HR 0.77, 95% CI 0.53–1.11), but the broad confidence intervals suggest that this study was not powered to detect a benefit in this group. Additionally, PEXIVAS did not mandate baseline biopsy data. We know AAV has a relapsing and remitting course which can lead to irreversible scarring of the kidneys. PR3-ANCA has a predominance towards more active lesions in the kidney, therefore a quicker rate of deterioration of renal function compared with the advanced fibrotic histology that can be observed in some subsets of MPO-ANCA [34]. The degree of fibrosis is also a predictor of poor renal outcome [35]. It is unknown to what proportion of the PEXIVAS cohort were unlikely to respond to PLEX due to chronic tubulointerstitial or glomerular damage. On the other hand, patients with an eGFR < 50 ml/min/1.73^2^ for longer than 3 months were excluded, which reduced the possibility of patients with chronic kidney damage being recruited.

From a pulmonary perspective, subgroup analysis of patients did not show any difference in effect. A small retrospective study demonstrated the benefit of PLEX in reducing mortality in DAH patients [36]. The French Vasculitis Study Group also showed that 96.7% of their 30 DAH patients in intensive care treated with PLEX recovered [27]. The PEXIVAS trial recruited 191 patients with pulmonary haemorrhage; of these, 61 were classified as severe (defined as oxygen saturation of < 85% or requiring mechanical ventilation). The hazard ratio of ESRF or death in those without haemorrhage was 0.95 (95% CI 0.69–1.31), non-severe haemorrhage was 0.64 (95% CI 0.33–1.24) and severe haemorrhage was 0.67 (95% CI 0.28–1.64). These findings could be interpreted as favourable towards DAH but are not conclusive. In reference to previous studies, important baseline characteristics and outcomes such as pulmonary function and its improvement, bronchoscopy findings and time to resolution of haemorrhage are not reported [36, 37]. As the composite outcomes were accounted for over the entire period of follow-up, this trial fails to capture any potential short-term benefit either. With only 9% of the entire cohort representing severe DAH and lack of relevant outcomes, PEXIVAS was likely not powered to detect the benefit of PLEX in this group. The findings of a retrospective cohort analysis at Mayo Clinic were congruent with PEXIVAS [37]. There were 73 AAV patients with DAH, of which 32 received PLEX and showed no significant differences in hospital mortality, length of hospital stay, duration of mechanical ventilation and remission at 6 months compared with those who did not receive PLEX. However, the intrinsic biases of open-label trials must be considered and PLEX was administered to sicker patients in this cohort. A further meta-analysis of mortality of DAH patients in AAV was unsuccessful in demonstrating a benefit of PLEX (odds ratio (OR) 0.41, 95% CI 0.0–4.50), although the wide confidence intervals and the substantial heterogeneity (*I*^2^ = 69%) bring the interpretation of the findings into question [24••].

Finally, there is a question of selection bias and ethical consideration to be raised. PEXIVAS aimed to recruit the critically ill amongst those with AAV. Physicians may feel reluctant to enrol their patients with an already high mortality risk to have the possibility of being randomised into not receiving PLEX or a lower glucocorticoid regimen.

Nevertheless, the findings of these studies have shaped the cornerstone of PLEX guidelines in treating severe AAV.

## Meta-analyses

In addition to the trials described above, three meta-analyses surrounding the efficacy of PLEX in AAV have been published since the conclusion of the PEXIVAS trial.

Yamada et al. [38••] reviewed four randomised controlled trials (RCT) (*n* = 827) to investigate the efficacy of PLEX in AAV but failed to find improvement in primary outcomes. There was no difference in overall mortality (RR 0.93, 95% CI 0.70–1.24) or mortality at 6 months (RR 0.54, 95% CI 0.21–1.38), 1 year (RR 0.71, 95% CI 0.27–1.86), 5 years (RR 1.00, 95% CI 0.60–1.68) and 10 years (RR 0.86, 95% CI 0.13–5.48). Clinical remission was significant at 1 month (RR 1.57, 95% CI 1.07–2.30) and 3 months (1.67, 95% CI 1.06–2.61). Although clinical remission was only reported in two studies and defined differently, the authors used 12 months as the representative value and found the effect was lost (RR 1.02, 95% CI 0.91–1.15). The composite of ESRF and death was non-significant (RR 0.97, 95% CI 0.80–1.18). However, a difference was detected when looking at the early post-treatment period in the development of ESRF. The PLEX group at 1 month and 3 months had a RR of 0.14 (95% CI 0.03–0.77), but for the overall follow-up period, the RR was 0.85 (95% CI 0.57–1.28). This must be interpreted in the context of PEXIVAS missing from the early follow-up analysis as no data was available at 3 months, as the Kaplan–Meier curve published by Walsh et al. [33] indicates early benefit is lost over time.

A meta-analysis including observational studies and RCTs (*n* = 1235) struggled to conclusively find a strong benefit of PLEX in AAV [6••]. The rates of mortality at 3 months (RR 0.79, 95% CI 0.19–3.25) and 12 months (RR 0.73, 95% CI 0.40–1.34) and ESRF at 3 months (RR 0.30, 95% CI 0.30–2.42) or 12 months (RR 1.32, 95% CI 0.53 3.25) were insignificant. This trend remained in disease relapse, incidence of infection and serious adverse events. Interestingly, time-to-event analyses revealed the overall incidence of ESRF was significantly lower in the PLEX group (HR 0.71, 95% CI 0.55–0.92) but not mortality (HR 0.96, 95% CI 0.72–1.29). This effect was lost once the MEPEX trial was excluded from the analysis.

Finally, Zhu and colleagues [24••] included 19 articles (5 RCTs and 14 cohort studies), totalling 1999 patients (PLEX = 886). There was no reduction in all-cause mortality at 3 months (OR 0.64, 95% CI 0.32–1.30), 1 year (OR 0.83, 95% CI 0.60–1.14), 5 years (OR 0.84, 95% CI 0.59–1.19) or end of follow-up (OR 1.18, 95% CI 0.78–1.79). The significant benefit of PLEX in reduction of ESRF at 3 months appears consistent with an OR of 0.32 (95% CI 0.16–0.66). No difference in adverse events, serious infections or ESRF rates were detected at 1 month, 6 months, 12 months, 5 years, or the end of follow-up.

In conclusion, the results of the meta-analyses are unanimous. When receiving PLEX, there is an apparent reduction in ESRF at 3 months without change in all-cause mortality. Despite the association between ESRF and mortality, the discrepancy could be partly accounted for by the introduction of rituximab and improvements in the quality of intensive care units. Previously, PLEX may have played a bigger role in salvaging renal function and therefore survival than it does now.

Serious adverse events, most importantly serious infections are another factor to consider. Previous hypotheses have speculated that the risk of increased infections with PLEX is due to attenuation of the immune system via the removal of immunoglobulins and complement proteins. Whilst some smaller studies have observed an increased incidence of serious infections, the above meta-analyses failed to detect an effect [39]. There is strong evidence that PLEX has similar rates of infection to standard induction treatment, but the nature and aetiology of these infections could be of great value [6••, 24••, 38••]. Most studies do not publish this data but if there is a possibility that the risk could be ameliorated then this could be a potential avenue in reducing mortality. For example, if the infections are related to the procedure of PLEX such as central venous access or bacterial pneumonia secondary to DAH then this could be somewhat anticipated and prevented.

In clinical practice, it is still reasonable to consider PLEX in those with either rapidly decreasing renal function or in the setting of DAH (Table 1).

**Table 1:** Summary of studies

Author (publication year)	Summary of findings
Jayne et al. (2007)	PLEX was found to have a beneficial effect on renal recovery at 3 and 12 months but this effect was lost at follow-up (median 3.95 years). No difference in patient survival
Walsh et al. (2020)	PLEX did not reduce the incidence of death or ESRF. However, this study had broader inclusion criteria than Jayne et al. and was likely not powered to detect a difference in the sickest patients (severe DAH and serum creatinine ≥ 500 umol/l)
Bellos et al. (2021)	4 RCTs and 5 retrospective studies (*n* = 1235) showed no benefit of PLEX in mortality or ESRF
Yamada et al. (2021)	4 RCTs (*n* = 827) showed no improvement in mortality. Early benefit of PLEX at 1 and 3 months detected
Zhu et al. (2021)	5 RCTs and 14 cohort studies (*n* = 1999) showed no reduction in mortality. Benefit of PLEX in reducing ESRF at 3 months was found

Research Agenda

• Cost–benefit and utility analysis of PLEX in the treatment of AAV

• Risk stratification models for ESRF in patients with AAV who might benefit the most from PLEX

• Clinical trials of PLEX in the setting of DAH

• Use of PLEX as rescue therapy in patients with AAV who have not responded to standard remission induction treatment

## Current Guidelines on PLEX

Despite the long-anticipated results from the PEXIVAS trial [33], the updated guidelines on the use of PLEX are equivocal. In 2020, the American Society of Apheresis (ASFA) downgraded the recommendation of the use of PLEX in rapidly progressive glomerulonephritis (RPGN) in the context of AAV from first-line to second-line therapy [40•]. The American College of Rheumatology (ACR) 2021 guidance for the management of AAV is conditionally opposed to the addition of PLEX in both active glomerulonephritis and alveolar haemorrhage [41•]. The European Alliance of Associations for Rheumatology formerly the European League Against Rheumatism (EULAR) and European Renal Association – European Dialysis and Transplant Association (ERA-EDTA) 2016 guidelines, published before results of PEXIVAS were known was based on findings of the MEPEX trial [31••] and a meta-analysis of PLEX for renal vasculitis and idiopathic rapidly progressive glomerulonephritis [23], recommended the consideration of PLEX in new or relapsing AAV if the patient’s serum creatinine is ≥ 500 umol/l due to RPGN (level of evidence 1B, grade of recommendation B) and to also consider PLEX in the context of severe DAH (level of evidence 3, grade of recommendation C) [42].

## Conclusion

The current evidence base shows no added long-term benefit of PLEX in addition to standard care in preventing ESRF or death. However, there is a possibility that PLEX delays dialysis dependence and ESRF in the early stages of the disease. Regardless of whether this is only for 3 to 12 months, this could be of clinical significance and a substantial improvement in the patient’s quality of life. Cost utility analysis and trials including patient-centred outcomes are required. Furthermore, ascertaining those at high risk of developing ESRF could help identify those who may benefit from PLEX the most.

The efficacy of PLEX in DAH still remains unanswered. Whilst some single-centre studies have demonstrated clinical improvement of DAH in AAV, other observational studies and subgroup analyses from randomised trials have failed to detect a benefit [27, 36, 37]. There remain fundamental limitations in current trials such as inadequacy of power and/or important primary outcome measures when considering the utility of PLEX in DAH. We know that the degree of hypoxaemia correlates to respiratory failure in these patients [37]. Objectively categorising the severity of DAH and their response to PLEX in a well-designed randomised trial would clarify this issue.

Patients with treatment-resistant AAV could be another cohort who might benefit from PLEX. De Joode et al. [8] demonstrated the potential use of PLEX as rescue therapy in those who had progressive renal failure despite standard induction, showing comparable rates of renal recovery at 6 months. Whilst we have no definitive answers for patients who remain critically ill despite glucocorticoids and immunosuppression, we cannot exclude PLEX from our armamentarium whilst we have no better alternatives in managing acutely life-threatening patients.

Despite the historical favouring of PLEX, the current evidence suggests that the role of PLEX in AAV may be limited. PEXIVAS may be a critical stepping stone in accepting that PLEX is not as efficacious as initially believed. However, certain subgroups may benefit and, for example, a well-designed trial with a larger sample of severe DAH patients is needed. This review confirms that PLEX should not be used in all patients with AAV. However, there may still be a benefit in certain subgroups and identification of these patients remains paramount.

## References

[1] Nelveg-Kristensen KE, Szpirt W, Carlson N, McClure M, Jayne D, Dieperink H (2020). Increasing incidence and improved survival in ANCA-associated vasculitis-a Danish nationwide study. Nephrol Dial Transplant.

[2] Jennette JC, Falk RJ, Bacon PA, Basu N, Cid MC, Ferrario F (2013). 2012 revised International Chapel Hill Consensus Conference Nomenclature of Vasculitides. Arthritis Rheum.

[3] Jennette JC, Nachman PH (2017). ANCA glomerulonephritis and vasculitis. Clin J Am Soc Nephrol.

[4] Specks U, Merkel PA, Seo P, Spiera R, Langford CA, Hoffman GS (2013). Efficacy of remission-induction regimens for ANCA-associated vasculitis. N Engl J Med.

[5] Little MA, Nightingale P, Verburgh CA, Hauser T, De Groot K, Savage C (2010). Early mortality in systemic vasculitis: relative contribution of adverse events and active vasculitis. Ann Rheum Dis.

[6.••] Bellos I, Michelakis I, Nikolopoulos D. The role of plasma exchange in antineutrophil cytoplasmic antibody-associated vasculitis: a meta-analysis. Clin Rheumatol. 2021;40(4):1447–56. 10.1007/s10067-020-05390-z. **Meta-analysis including PEXIVAS which demonstrated no improvement in long-term renal dependence or all-cause mortality.** 10.1007/s10067-020-05390-z 32935248

[7] Titeca-Beauport D, Francois A, Lobbedez T, Guerrot D, Launay D, Vrigneaud L (2018). Early predictors of one-year mortality in patients over 65 presenting with ANCA-associated renal vasculitis: a retrospective, multicentre study. BMC Nephrol.

[8] de Joode AA, Sanders JS, Smid WM, Stegeman CA (2014). Plasmapheresis rescue therapy in progressive systemic ANCA-associated vasculitis: single-center results of stepwise escalation of immunosuppression. J Clin Apher.

[9] Bobati SS, Naik KR. Therapeutic plasma exchange - an emerging treatment modality in patients with neurologic and non-neurologic diseases. J Clin Diagn Res. 2017;11(8):EC35-EC7. 10.7860/JCDR/2017/27073.10480. 10.7860/JCDR/2017/27073.10480 PMC5620780 28969140

[10] Padmanabhan A, Connelly-Smith L, Aqui N, Balogun RA, Klingel R, Meyer E, et al. Guidelines on the use of therapeutic apheresis in clinical practice - evidence-based approach from the writing committee of the American Society for Apheresis: The Eighth Special Issue. J Clin Apher. 2019;34(3):171–354. 10.1002/jca.21705. 10.1002/jca.21705 31180581

[11] Mokrzycki MH, Kaplan AA (1994). Therapeutic plasma exchange: complications and management. Am J Kidney Dis.

[12] Chevret S, Hughes RA, Annane D. Plasma exchange for Guillain-Barre syndrome. Cochrane Database Syst Rev. 2017;2:CD001798. 10.1002/14651858.CD001798.pub3. 10.1002/14651858.CD001798.pub3 PMC6464100 28241090

[13] Apheresis in the office setting. J Oncol Pract. 2008;4(2):94–5.10.1200/JOP.0817002 10.1200/JOP.0817002 PMC2793967 20856786

[14] Nakazawa D, Masuda S, Tomaru U, Ishizu A (2019). Pathogenesis and therapeutic interventions for ANCA-associated vasculitis. Nat Rev Rheumatol.

[15] Lamprecht P, Kerstein A, Klapa S, Schinke S, Karsten CM, Yu X (2018). Pathogenetic and clinical aspects of anti-neutrophil cytoplasmic autoantibody-associated vasculitides. Front Immunol.

[16] Csernok E (2003). Anti-neutrophil cytoplasmic antibodies and pathogenesis of small vessel vasculitides. Autoimmun Rev.

[17] Csernok EMA, Gross WL (1999). Immunopathology of ANCA-associated vasculitis. Int Med.

[18] Frausova D, Hruskova Z, Lanska V, Lachmanova J, Tesar V (2016). Long-term outcome of patients with ANCA-associated vasculitis treated with plasma exchange: a retrospective, single-centre study. Arthritis Res Ther.

[19] Xiao H, Heeringa P, Hu P, Liu Z, Zhao M, Aratani Y (2002). Antineutrophil cytoplasmic autoantibodies specific for myeloperoxidase cause glomerulonephritis and vasculitis in mice. J Clin Investig.

[20] Xiao H, Heeringa P, Liu Z, Huugen D, Hu P, Maeda N (2005). The role of neutrophils in the induction of glomerulonephritis by anti-myeloperoxidase antibodies. Am J Pathol.

[21] Little MA, Smyth L, Salama AD, Mukherjee S, Smith J, Haskard D (2009). Experimental autoimmune vasculitis: an animal model of anti-neutrophil cytoplasmic autoantibody-associated systemic vasculitis. Am J Pathol.

[22] Tomasson G, Grayson PC, Mahr AD, Lavalley M, Merkel PA (2012). Value of ANCA measurements during remission to predict a relapse of ANCA-associated vasculitis–a meta-analysis. Rheumatology (Oxford).

[23] Walsh M, Catapano F, Szpirt W, Thorlund K, Bruchfeld A, Guillevin L (2011). Plasma exchange for renal vasculitis and idiopathic rapidly progressive glomerulonephritis: a meta-analysis. Am J Kidney Dis.

[24.••] Zhu Y, Rao J, Liu L, Ou J, Li W, Xue C. The therapeutic effect of plasma exchange on ANCA-associated vasculitis: a meta-analysis. Clin Nephrol. 2021;95(6):312–22. 10.5414/CN110410. **Meta-analysis including PEXIVAS which demonstrated no improvement in long-term renal dependence or all-cause mortality.** 10.5414/CN110410 33749583

[25] Finkielman JDMP, Schroeder D (2007). Antiproteinase 3 antineutrophil cytoplasmic antibodies and disease activity in Wegener granulomatosis. Ann Intern Med.

[26] Cortazar FB, Niles JL (2020). The fate of plasma exchange and glucocorticoid dosing in ANCA-associated vasculitis after PEXIVAS. Am J Kidney Dis.

[27] de Luna G, Chauveau D, Aniort J, Carron PL, Gobert P, Karras A (2015). Plasma exchanges for the treatment of severe systemic necrotizing vasculitides in clinical daily practice: data from the French Vasculitis Study Group. J Autoimmun.

[28] Pusey CD, Ress AJ, Evans DJ, Peters DK, Lockwood CM (1991). Plasma exchange in focal necrotizing glomerulonephritis without anti-GBM antibodies. Kidney Int..

[29] Saal SD, Gordon B, Evans E, Stack J, Suthanthiran M (1981). Plasmapheresis in the prevention and treatment of rapidly progressive renal disease. Clin Exp Dial Apheresis.

[30] Bruns FJ, Adler S, Fraley DS, Segel DP (1989). Long-term follow-up of aggressively treated idiopathic rapidly progressive glomerulonephritis. Am J Med.

[31.••] Jayne DR, Gaskin G, Rasmussen N, Abramowicz D, Ferrario F, Guillevin L, et al. Randomized trial of plasma exchange or high-dosage methylprednisolone as adjunctive therapy for severe renal vasculitis. J Am Soc Nephrol. 2007;18(7):2180–8. 10.1681/ASN.2007010090. **First large scale randomised trial which detected early beneficial effect of PLEX in AAV.** 10.1681/ASN.2007010090 17582159

[32.••] Walsh M, Casian A, Flossmann O, Westman K, Hoglund P, Pusey C, et al. Long-term follow-up of patients with severe ANCA-associated vasculitis comparing plasma exchange to intravenous methylprednisolone treatment is unclear. Kidney Int. 2013;84(2):397–402. 10.1038/ki.2013.131. **Most recent and largest randomised trial demonstrating no significant benefit of PLEX in AAV management.** 10.1038/ki.2013.131 23615499

[33] Walsh M, Merkel PA, Peh CA, Szpirt WM, Puechal X, Fujimoto S (2020). Plasma exchange and glucocorticoids in severe ANCA-associated vasculitis. N Engl J Med.

[34] Franssen CF, Gans RO, Arends B, Hageluken C, ter Wee PM, Gerlag PG (1995). Differences between anti-myeloperoxidase- and anti-proteinase 3-associated renal disease. Kidney Int.

[35] de Lind van Wijngaarden RA, Hauer HA, Wolterbeek R, Jayne DR, Gaskin G, Rasmussen N, et al. Clinical and histologic determinants of renal outcome in ANCA-associated vasculitis: a prospective analysis of 100 patients with severe renal involvement. J Am Soc Nephrol. 2006;17(8):2264–74. 10.1681/ASN.2005080870. 10.1681/ASN.2005080870 16825335

[36] Klemmer PJ, Chalermskulrat W, Reif MS, Hogan SL, Henke DC, Falk RJ (2003). Plasmapheresis therapy for diffuse alveolar hemorrhage in patients with small-vessel vasculitis. Am J Kidney Dis.

[37] Cartin-Ceba R, Diaz-Caballero L, Al-Qadi MO, Tryfon S, Fervenza FC, Ytterberg SR (2016). Diffuse alveolar hemorrhage secondary to antineutrophil cytoplasmic antibody-associated vasculitis: predictors of respiratory failure and clinical outcomes. Arthritis rheumatol.

[38.••] Yamada Y, Harada M, Hara Y, Iwabuchi R, Hashimoto K, Yamamoto S, et al. Efficacy of plasma exchange for antineutrophil cytoplasmic antibody-associated systemic vasculitis: a systematic review and meta-analysis. Arthritis Res Ther. 2021;23(1):28. 10.1186/s13075-021-02415-z. **Meta-analysis including PEXIVAS which demonstrated no improvement in long-term renal dependence or all-cause mortality.** 10.1186/s13075-021-02415-z PMC7809754 33446268

[39] Solar-Cafaggi D, Atisha-Fregoso Y, Hinojosa-Azaola A (2016). Plasmapheresis therapy in ANCA-associated vasculitides: a single-center retrospective analysis of renal outcome and mortality. J Clin Apheresis.

[40.•] Balogun RA, Sanchez AP, Klingel R, Witt V, Aqui N, Meyer E, et al. Update to the ASFA guidelines on the use of therapeutic apheresis in ANCA-associated vasculitis. J Clin Apheresis. 2020;35(5):493–9. 10.1002/jca.21820. **Updated guidance from ASFA acknowledging the new evidence and downgrading recommendations.** 10.1002/jca.21820 32770558

[41.•] Chung SA, Langford CA, Maz M, Abril A, Gorelik M, Guyatt G, et al. 2021 American College of Rheumatology/Vasculitis Foundation Guideline for the Management of Antineutrophil Cytoplasmic Antibody-Associated Vasculitis. Arthritis rheumatol. 2021;73(8):1366–83. 10.1002/art.41773. **Updated guidance from ACR acknowledging new evidence and conditionally not recommending PLEX in AAV.** 10.1002/art.41773 PMC12327957 34235894

[42] Yates M, Watts RA, Bajema IM, Cid MC, Crestani B, Hauser T (2016). EULAR/ERA-EDTA recommendations for the management of ANCA-associated vasculitis. Ann Rheum Dis.

